# Tidal Volume Discrepancy for Air Leak Detection in Thoracoscopic Lung Resection in Smokers

**DOI:** 10.1016/j.atssr.2026.02.028

**Published:** 2026-03-11

**Authors:** Masaya Otabe, Masayuki Ishida, Atsushi Kamigaichi, Atsushi Kagimoto, Michiyoshi Sanuki, Takeshi Mimura

**Affiliations:** 1 Department of General Thoracic Surgery, National Hospital Organization, Kure Medical Center and Chugoku Cancer Center, Kure, Hiroshima, Japan; 2 Department of Anesthesiology, National Hospital Organization, Kure Medical Center and Chugoku Cancer Center, Kure, Hiroshima, Japan

## Abstract

**Background:**

Prolonged air leakage (PAL) is a common postoperative complication after lung resection. The conventional air leak test, widely used to detect intraoperative air leaks, is limited to restricted thoracic space during lung inflation, which may result in undetected leaks, and fragile lung parenchyma may allow new air leaks to develop during chest closure. This study evaluated the efficacy of tidal volume discrepancy monitoring for intraoperative air leak detection during video-assisted thoracoscopic surgery anatomical lung resection (lobectomy and segmentectomy).

**Methods:**

We retrospectively reviewed 310 patients with a smoking history who underwent video-assisted thoracoscopic surgery anatomical lung resection for non-small cell lung cancer. Since May 2020, a new intraoperative air leak assessment method was implemented. After the conventional air leak test, bilateral lung ventilation was continued until chest closure, with continuous monitoring of inspired and expired tidal volumes. A sustained discrepancy >20 mL per breath prompted further intrathoracic inspection and repair.

**Results:**

PAL occurred in 4.1% of patients in the new method group, significantly lower than 10.9% in the conventional method group (*P* = .0205). Multivariate analysis identified the evaluation method and presence of intrathoracic adhesion as independent predictors of PAL. Importantly, all procedures were completed thoracoscopically, including a substantial number of segmentectomies.

**Conclusions:**

Intraoperative evaluation of intraoperative air leaks using tidal volume discrepancies is a reliable and effective method to detect air leaks and reduce PAL incidence in video-assisted thoracoscopic surgery anatomical lung resections.

## Visual Abstract

**Figure:**
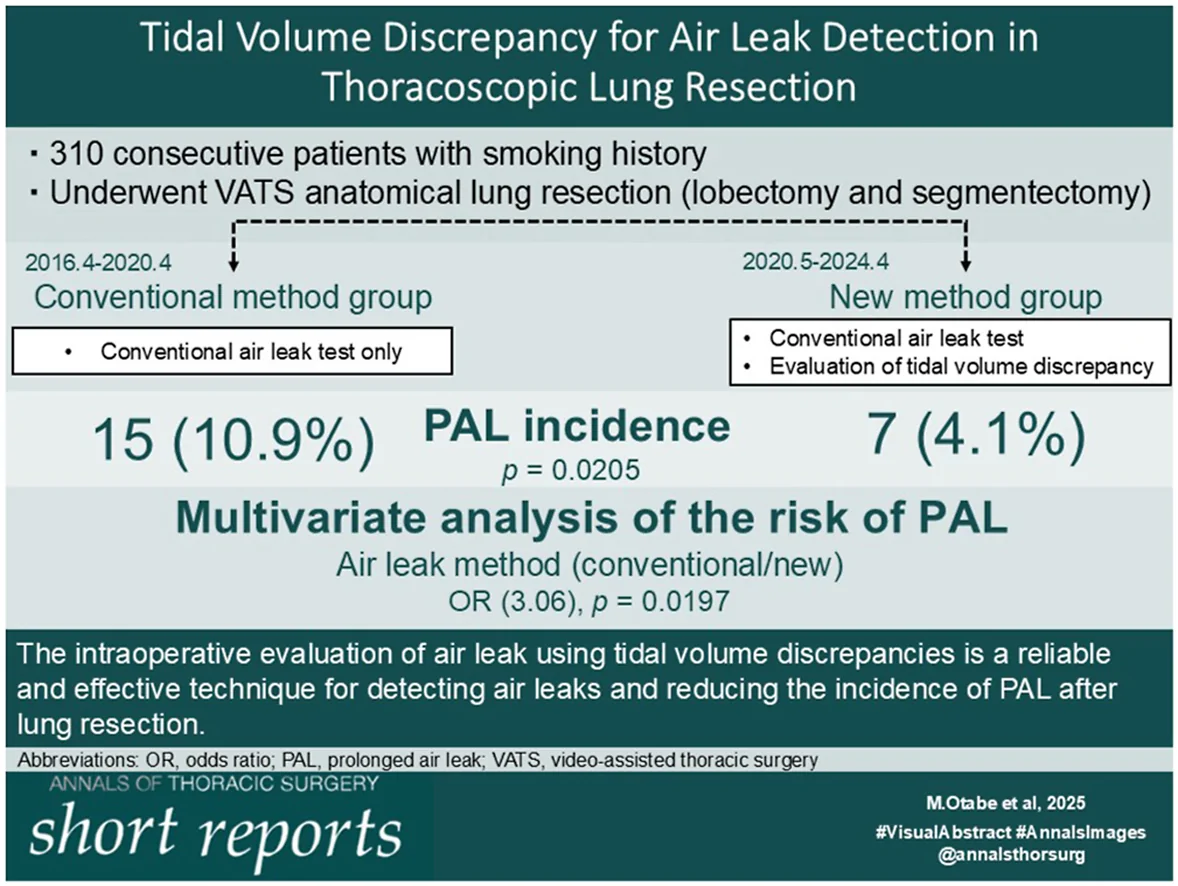

**In Short**

▪ Missed intraoperative air leaks can cause prolonged air leakage (PAL) after thoracoscopic lung resection.
▪ The conventional air leak test is limited by poor visualization and subjectivity.
▪ Intraoperative evaluation of tidal volume discrepancies is a reliable and effective technique for detecting air leaks and reducing PAL after lung resection.

Prolonged air leakage (PAL), a major postoperative complication of lung resection, leads to several conditions such as empyema.^1^ The air leak test is a commonly used method for evaluating intraoperative air leaks (IALs) by detecting air bubbles in fluid. However, the test requires lung inflation, which reduces the thoracic space and creates blind spots during video-assisted thoracoscopic surgery (VATS), potentially resulting in missed air leaks. Furthermore, fragile lung parenchyma may allow new air leaks to develop during chest closure.

Brunelli and associates^2^ reported the efficacy of an alternative IAL evaluation method that entails measuring the difference between the inspired and expired volumes in ventilation parameters to predict postoperative PAL after lobectomies. However, 33 of 111 patients (29.7%) underwent VATS lobectomy in their study, which does not adequately reflect contemporary realities as VATS is now the predominant approach for lung resection. Meanwhile, the importance of lung segmentectomy for cases of small-sized lung cancer has been increasing in recent years.^3^ During the air leak test, it is more likely to miss air leaks in lung segmentectomy, which has a smaller dead space.

This study aimed to assess the efficacy of evaluating the difference in ventilation parameters between inspired and expired tidal volumes (TVs) for detecting IAL and predicting postoperative air leaks in patients who underwent VATS anatomical lung resection, including lobectomy and segmentectomy. We specifically focused on patients with a smoking history, a major risk factor for PAL.^4^

## Patients and Methods

### Patients

We retrospectively analyzed a prospectively collected database of 310 consecutive patients with a smoking history who underwent VATS anatomical lung resection (lobectomy and segmentectomy) for non-small cell lung cancer between April 2016 and April 2024 at the National Hospital Organization Kure Medical Center and Chugoku Cancer Center. Patients without a smoking history and those who underwent wedge resection, pneumonectomy, lung resection under thoracotomy, or conversion from VATS to thoracotomy were excluded. Preoperative assessments were performed according to institutional protocols (details provided in the Supplemental Methods). Our institutional review board approved this study and waived the informed consent requirement (approval no. 2020-11).

### Surgical Procedure and Air Leak Evaluation

**Figure 1:**
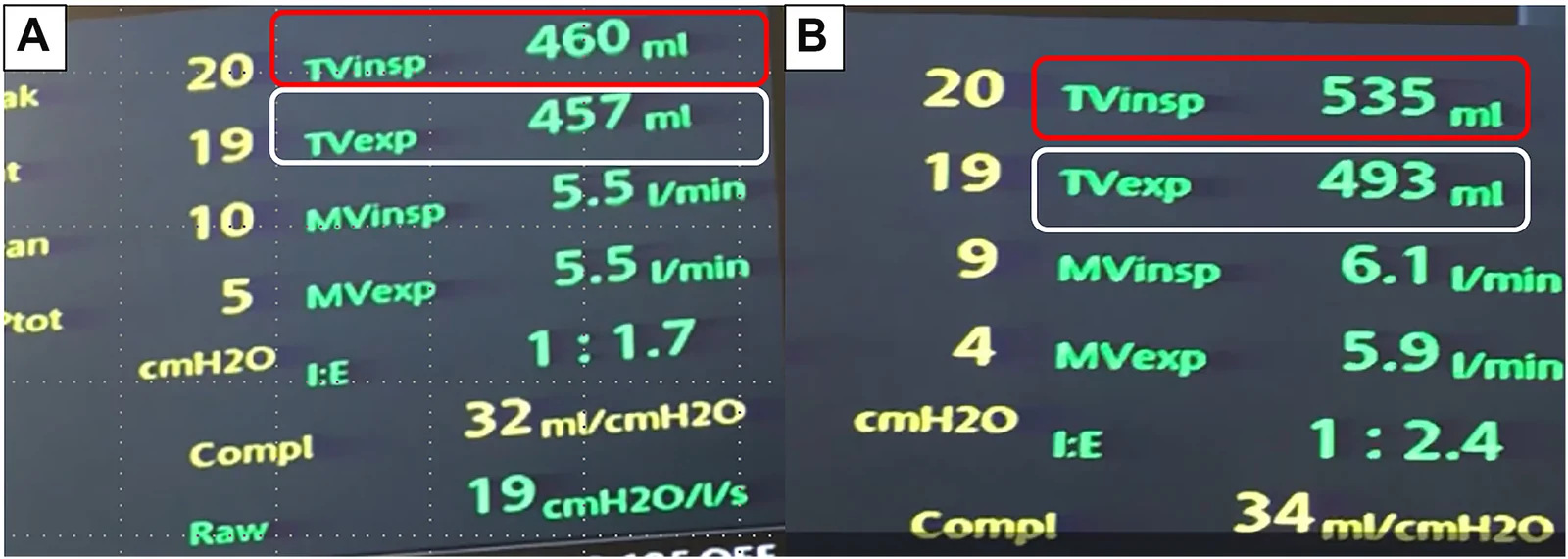
Ventilator parameters for intraoperative air leak assessment. Red and white circles indicate inspired and expired tidal volumes (TVs), respectively. (A) Case with TV difference ≤20 mL per ventilation. (B) Case with TV difference >20 mL per ventilation.

All resections were performed via VATS. Pulmonary vessels, bronchus, and interlobar or intersegmental fissures were divided using a surgical stapler. Intrathoracic adhesions were classified as none (absent or localized) or present (widespread). After resection, the conventional air leak test was performed with an airway pressure of 20 cm H_2_O. Detected leaks were repaired using soft coagulation, sutures, or reinforcement with fibrin glue and a polyglycolic acid sheet. Since May 2020, we also measured TV discrepancies under stable bilateral ventilation at 20 cm H_2_O (Figure 1). Importantly, this assessment was performed continuously and repeatedly throughout the procedure until chest closure. A difference of >20 mL per ventilation was considered suggestive of air leak and prompted thorough thoracic reinspection and, when necessary, immediate repair (Supplemental Figure 1). Ventilator settings are described in Supplemental Methods. Postoperative management included chest tube placement and suction with an electronic drainage device at –7 cm H_2_O.

PAL was defined as an air leak persisting >5 days. Additional treatment included pleurodesis, reoperation, or chest tube reinsertion. Patients evaluated only by air leak formed the conventional group; those assessed with the TV method formed the new method group. Statistical analyses were performed as described in Supplemental Methods.

## Results

Supplemental Table 1 summarizes participant characteristics of the entire cohort (N = 310). Median age was 72 years (interquartile range, 67-77 years), 87.1% were male, and 35.8% underwent segmentectomy. Intrathoracic adhesions occurred in 16.8%. Median chest tube duration was 2 days. Overall, PAL occurred in 7.1%, and 11.0% required additional postoperative air leak treatment.

Compared with the conventional group, patients in the new method group were older (median 73 vs 71 years, *P* = .007) and more frequently underwent segmentectomy (45.9% vs 23.2%, *P* < .001) (Supplemental Table 2). In the new method group, 5 patients demonstrated a difference greater than 20 mL per ventilation. In these cases, we reexplored the thoracic cavity and performed successful intraoperative repairs. These air leaks were not detected by the conventional air leak test. The sites of the air leaks and the repair methods used are presented in Supplemental Table 3. The incidence of PAL was lower in the new method group (4.1% vs 10.9%, *P* = .020), and chest tube duration was shorter (median 2 vs 2 days, *P* = .019) (Table 1). Additional treatment tended to be lower in the new method group, though not statistically significant (8.1% vs 14.5%, *P* = .075).

**Table 1:** Comparison of Perioperative Outcomes Between the Conventional and New Evaluation Methods

Variables	Conventional Group (n = 138)	New Group (n = 172)	*P* Value
Adhesion	26 (18.8)	26 (15.1)	.3831
Operation time, min			**.0299**
Median (IQR)	218 (188-245)	208 (170-240)	
Mean (SD)	221 (50.6)	207 (46.9)	
Duration of chest tube placement, days			**.0193**
Median (IQR)	2 (2-3)	2 (2-3)	
Mean (SD)	3.4 (3.4)	2.6 (1.7)	
Occurrence of prolonged air leak	15 (10.9)	7 (4.1)	**.0205**
Additional treatment for air leaks	20 (14.5)	14 (8.1)	.0752
Pleurodesis	16 (11.6)	8 (4.7)	**.0230**
Reoperation	5 (3.6)	1 (0.6)	.0534
Reinsertion or exchange of a chest tube	6 (4.4)	7 (4.1)	.9034

Values are presented as n (%) unless otherwise noted. Boldface highlights significant differences.

IQR, interquartile range; SD, standard deviation.

Univariate analysis identified evaluation method (odds ratio [OR], 2.87; 95% CI, 1.14-7.26; *P* = .026) and intrathoracic adhesions (OR, 3.17; 95% CI, 1.26-8.00; *P* = .0146) as predictors of PAL. Multivariate analysis confirmed both as independent risk factors (evaluation method: OR, 2.78; 95% CI, 1.09-7.08; *P* = .032; adhesions: OR, 3.04; 95% CI, 1.19-7.76; *P* = .020) (Supplemental Table 4). Comparisons according to surgical procedure are shown in the Supplemental Results and presented in Supplemental Table 5.

## Comment

This study demonstrated that incorporating a new IAL evaluation method based on TV discrepancies in ventilator parameters notably reduced the incidence of PAL after VATS anatomical lung resection compared to the conventional air leak method alone. Because smoking-related emphysematous changes predispose patients to PAL, we focused on those with a smoking history since they represent a high-risk population.^4^ Segmentectomy, a technique in which the smaller residual thoracic cavity can create blind spots during the air leak test, was performed, potentially leading to missed air leaks. By addressing these challenges, this study provides valuable insights into IAL management optimization during VATS anatomical lung resection.

PAL, a major postoperative complication of lung resection, leads to other complications such as empyema.^1^ Additional treatment for air leaks, such as reoperation, drain replacement, or pleurodesis, significantly burdens patients.^4^ Previous studies have identified smoking, low pulmonary function, and intrathoracic adhesion as risk factors for PAL; however, research on PAL prevention strategies in high-risk patients remains limited.^5^ Additionally, while various reports have explored intraoperative and perioperative preventive measures, such as fibrin glue, surgical staplers,^6^^,^^7^ relatively little research has been conducted on IAL evaluation methods. The conventional method for evaluating air leaks is the air leak test, which detects air bubbles in distilled water or saline in an inflated lung after lung resection. This widely used method has several limitations. The inflated lung reduces the available intrathoracic space, creating blind spots, especially during VATS. Moreover, the subjective nature of air bubble volume assessment leads to inconsistencies in the evaluation of the degree of air leak among surgeons.

Although the air leak test remains the standard for IAL detection, its subjectivity and limited visualization in VATS necessitate alternative approaches. Our ventilator-based method provides objective quantification and minimizes intersurgeon variability. Brunelli and colleagues^2^ first proposed an alternative IAL evaluation method that entails determining the difference between the inspired and expired TVs. However, their study included both thoracotomy and VATS cases, with a relatively small number of patients undergoing VATS lung resection. Messina and associates^8^ also reported the efficacy of a similar method in VATS; but only in lobectomy cases. We conducted a study on VATS anatomical lung resection, including segmentectomy, whose demand has been on the rise.^3^ Particularly in thoracoscopic surgery, the more lung parenchyma that remains (as in lung segmentectomy), the more blind spots are created, increasing the likelihood of missing air leaks during the IAL test. Initially, we aimed to identify the difference between the inspired and expired TVs that could predict PAL through a prospective observational study. However, in cases where such a difference was detected, additional thorough closure procedures for IAL were performed. In one case, a TV discrepancy detected during chest closure led to reexploration, revealing a dehiscence at the stapler resection line. This early detection enabled timely intervention and prevented complications (see Supplemental Video). Consequently, we did not observe findings similar to those of Brunelli and coworkers, who reported that an IAL > 500 mL/min was associated with an increased risk of postoperative complications. As a result, we compared cases before and after the introduction of this IAL evaluation method using a historical control group (the conventional method group). Our findings demonstrated a significant reduction in PAL in the new method group. The rationale for setting the threshold at a TV difference of 20 mL per ventilation when suspecting an air leak is based on the following considerations. Brunelli and associates^2^ identified a TV difference of 500 mL per minute as a predictor of PAL. With a respiratory rate of 12 breaths per minute, this corresponds to approximately 40 mL per ventilation. Based on our clinical experience, we aimed to minimize the TV difference to around 20 mL per ventilation whenever possible. Although the additional step might theoretically prolong the surgical procedure, our data indicate that the procedure did not last longer with the new evaluation method than with the conventional evaluation method. This suggests that the new evaluation method can be performed easily and without prolonging the surgical procedure. Furthermore, as a reproducible evaluation method, it minimizes intersurgeon variability, ensuring consistent and reliable air leak detection.

Nevertheless, this study had several limitations. First, it was a single-center retrospective study. There is a possibility of selection bias as the new method was only introduced in 2020. Second data on the number of patients with IAL in air leak test and details of their repairs, including method used, were not available. Despite these limitations, our findings indicate that integrating this new evaluation method with the conventional air leak test has clearly contributed to a reduction in the incidence of PAL, particularly in high-risk patients with a history of smoking. This study included patients who underwent segmentectomy, allowing evaluation of a broader range of VATS anatomical lung resections. Although robotic-assisted thoracic surgery was not included in this study, our method may also be applicable to robotic procedures. The findings highlight the clinical utility of this simple and reproducible method and its potential to improve perioperative outcomes after lung resection.

In conclusion, IAL evaluation by comparing inspired and expired TVs based on ventilation parameters is a reliable and effective air leak assessment method after lung surgery. This approach not only reduces the risk of PAL but also enhances intraoperative decision-making, suggesting its potential as a new standard in thoracic surgery.
