# Supplementary material for: Tidal Volume Discrepancy for Air Leak Detection in Thoracoscopic Lung Resection in Smokers
Source: Ann Thorac Surg Short Rep. 2026 Mar 11;4(3):909–13. doi: 10.1016/j.atssr.2026.02.028 (PMC13555444; doi:10.1016/j.atssr.2026.02.028)
Supplement: Supplementary file 1 [file mmc2.docx]

**Supplementary Materials**

**Supplementary Methods:**

**Preoperative Examinations**

Preoperative assessments were conducted with all patients to determine the clinical stage and appropriate treatment strategies. These included chest computed tomography (CT), whole-body ^18^F-fluorodeoxyglucose positron emission tomography/ computed tomography (FDG-PET/CT), brain magnetic resonance imaging (MRI), and pulmonary function tests. Each patient’s clinical stage was defined according to the eighth edition of the TNM Classification of Malignant Tumors.^1^

**Ventilator Settings and Assessment Method**

Ventilator settings were standardized as follows: a tidal volume of 7–8 mL/kg (adjusted for individual lung compliance), a respiratory rate of 10 breaths/min, and a positive end-expiratory pressure of 5 cm H₂O. After full re-expansion of the isolated lung, all measurements were obtained using the same ventilator, and the values were calculated automatically. We defined an air leak as a reproducible tidal volume discrepancy >20 mL/ breath during repeated ventilation. Tidal volumes were continuously monitored for discrepancies throughout the procedure until chest closure. In cases of tidal volume discrepancy in which no air leak was detected by the conventional air leak test and no apparent intraoperative cause of air leakage was apparent, alternative non-surgical factors were carefully considered. These included air leakage around the endotracheal tube cuff. Close communication with the anesthesiology team was essential to exclude such possibilities. If no cause could be identified, chest closure was continued with careful ongoing monitoring of tidal volume discrepancies.

**Statistical Analysis**

Continuous variables were presented as medians and interquartile ranges (IQRs) and categorical variables as frequencies and percentages. Continuous and categorical variables were analyzed using c the Mann–Whitney U test and the χ^2^ test, respectively. To identify independent risk factors for PAL, multivariate logistic regression analysis was performed using variables found to significantly predict PAL in univariate analyses JMP version 14 (SAS Institute, USA) was used for all statistical analyses, P-values <.05 were considered statistically significant.

**Supplementary Results**

Supplementary Table 5 provides a comparison of the patient characteristics and perioperative outcomes of those who underwent segmentectomy versus those who underwent lobectomy. Segmentectomy patients tended to be older (median 73 vs. 72 years, P = .057), and had a significantly lower median vital capacity (92.4 vs. 99.1 %, P = .003). There were no significant differences in chest tube duration (median 2 vs. 2 days, P = .130) or in the incidence of prolonged air leakage (4.5% vs. 8.5%, P = .273).

**References**

1. Goldstraw P, Chansky K, Crowley J, et al. The IASLC lung cancer staging project: proposals for revision of the TNM stage groupings in the forthcoming (eighth) edition of the TNM classification for lung cancer. *J Thorac Oncol*. 2016;11:39-51.

**Supplementary Tables:**

**Table S1. Patient characteristics**

| Variables | N = 310 |
| --- | --- |
| Age, years |  |
| Median (IQR) | 72 (67–77) |
| Mean (SD) | 71 (8.1) |
| Sex (male), N (%) | 270 (87.1) |
| Smoking (Brinkman Index) |  |
| Median (IQR) | 840 (500–1120) |
| Mean (SD) | 883 (574) |
| Method, N (%) |  |
| Segmentectomy | 111 (35.8) |
| Lobectomy | 199 (64.2) |
| Goddard score, N (%) |  |
| Normal or mild (0–7) | 237 (76.5) |
| Moderate (8–15) | 58 (18.7) |
| Severe (16–24) | 15 (4.8) |
| %VC, % |  |
| Median (IQR) | 96 (84.3–108) |
| Mean (SD) | 96 (17.8) |
| FEV1%, % |  |
| Median (IQR) | 76.2 (69.3–81.4) |
| Mean (SD) | 74 (10.5) |
| Presence of adhesion, N (%) | 52 (16.8) |
| Operation time, min |  |
| Median (IQR) | 210 (179–243) |
| Mean (SD) | 213 (48.9) |
| Duration of chest tube placement, days |  |
| Median (IQR) | 2 (2–3) |
| Mean (SD) | 2.7 (2.9) |
| Occurrence of prolonged air leakage, N (%) | 22 (7.1) |
| Additional treatment for air leaks, N (%) | 34 (11.0) |
| Pleurodesis | 24 (7.7) |
| Reoperation | 6 (1.9) |
| Reinsertion or exchange of the chest tube | 13 (4.2) |
| FEV1, forced expiratory volume in 1 second; IQR, interquartile range; SD, standard deviation; VC, vital capacity | |

**Table S2. Comparison of patient variables in the groups assessed for air leaks during thoracoscopic lung resection by the conventional or new method**

| Variables | Conventional group (N = 138) | New group (N = 172) | *p*-value | |
| --- | --- | --- | --- | --- |
| Age, years |  |  | 0.007 | |
| Median (IQR) | 71 (66–75) | 73 (68–77) |  | |
| Mean (SD) | 69.9 (7.8) | 72 (8.2) |  | |
| Sex (male), N (%) | 122 (88.4) | 148 (90.6) | 0.538 | |
| Brinkman Index, N |  |  | 0.083 | |
| Median (IQR) | 800 (508–1080) | 893 (500–1200) |  | |
| Mean (SD) | 806 (486) | 945 (631) |  | |
| Surgical method, N (%) |  |  | <0.001 | |
| Segmentectomy | 32 (23.2) | 79 (45.9) |  | |
| Lobectomy | 106 (76.8) | 93 (54.1) |  | |
| Goddard score, N (%) |  |  | 0.355 | |
| Normal or mild (0–7) | 107 (77.5) | 130 (75.6) |  | |
| Moderate (8–15) | 27 (19.6) | 31 (18.0) |  | |
| Severe (16–24) | 4 (2.9) | 11 (6.4) |  | |
| %VC, % |  |  | 0.0120 | |
| Median (IQR) | 101 (85.5–110) | 94.4 (83.9–104) |  | |
| Mean (SD) | 98 (18.3) | 94 (17.2) |  | |
| FEV1%, % |  |  | 0.234 | |
| Median (IQR) | 77.0 (70.7–81.6) | 75.4 (67.6–80.7) |  | |
| Mean (SD) | 75 (9.2) | 73 (11.4) |  | |
| FEV1, forced expiratory volume in 1 second; IQR, interquartile range; SD, standard deviation; VC, vital capacity | | | |  |

**Table S3. Clinical details of the re-exploration and repair methods used in thoracoscopic lung resection patients with detected air leaks**

| Variable | N = 5 |
| --- | --- |
| Surgical method, n (%) |  |
| Segmentectomy | 2 |
| Lobectomy | 3 |
| Air leak site, n (%) |  |
| Adhesiolysis site | 2 |
| Staple line | 3 |
| Air leak repair method* |  |
| Suture | 2 |
| Soft coagulation | 5 |
| Fibrin glue | 4 |
| Polyglycolic acid sheet | 3 |
| Pericardial fat pad | 4 |
| Additional resection | 1 |
| TachoSil® | 3 |

*The total number exceeds the number of patients, as more than one repair method was used in some patients.

**Table S4. Univariate and multivariate analyses of factors associated with prolonged air leaks in** **thoracoscopic lung resection patients**

|  | OR (95% CI) | *p*-value | OR (95% CI) | *p-*value |
| --- | --- | --- | --- | --- |
| Surgical method (lobectomy/ segmentectomy) | 1.98 (0.71–5.52) | 0.192 |  |  |
| Sex (male/ female) | 3.29 (0.43–25.15) | 0.251 |  |  |
| Goddard score (≥6/ <6) | 2.05 (0.85–4.95) | 0.110 |  |  |
| %VC (≥80/ <80) | 1.30 (0.37–4.56) | 0.684 |  |  |
| FEV1% (≥70/ <70) | 0.64 (0.26–1.58) | 0.333 |  |  |
| Age (≥70/ <70) | 1.81 (0.65–5.05) | 0.258 |  |  |
| Adhesion (presence/ absence) | 3.18 (1.26–8.03) | 0.015 | 3.36 (1.32–8.59) | 0.011 |
| Air leak test method (conventional/ new) | 2.87 (1.14–7.26) | 0.026 | 3.06 (1.20–7.81) | 0.020 |
| CI, confidence interval; FEV1, Forced expiratory volume in 1 second; OR, odds ratio; VC, vital capacity | | | | |

| Table S5. Comparison of patient characteristics and perioperative outcomes between patients who underwent segmentectomy and lobectomy | | | | |
| --- | --- | --- | --- | --- |
| Variables | **Segmentectomy (N=111)** | **Lobectomy (N=199)** | ***p*-value** | |
| Age, years |  |  | 0.057 | |
| Median (IQR) | 73 (68.5–77) | 72 (66–76) |  | |
| Mean (SD) | 72.4 (7.6) | 70.4 (8.3) |  | |
| Sex (male), N (%) | 95 (85.6) | 175 (87.9) | 0.677 | |
| Brinkman Index |  |  | 0.760 | |
| Median (IQR) | 840 (433–1140) | 840 (523–1100) |  | |
| Mean (SD) | 904 (664) | 871 (519) |  | |
| Goddard score, N (%) |  |  | 0.605 | |
| Nomal or mild (0–7) | 85 (76.6) | 152 (76.4) |  | |
| Moderate (8–15) | 19 (17.1) | 39 (19.6) |  | |
| Severe (16–24) | 7 (6.3) | 8 (4.0) |  | |
| %VC, % |  |  | 0.003 | |
| Median (IQR) | 92.4 (81.1–105.7) | 99.1 (88.5–109.3) |  | |
| Mean (SD) | 92.3 (20.4) | 98.5 (15.8) |  | |
| FEV1%, % |  |  | 0.676 | |
| Median (IQR) | 76.3 (68.3–81.5) | 76.1 (70.1–81.0) |  | |
| Mean (SD) | 73.5 (11.1) | 74.5 (10.2) |  | |
| Adhesion, N (%) | 19 (17.1) | 33 (16.6) | 1 | |
| Operation time, min |  |  | 0.188 | |
| Median (IQR) | 208 (176–235) | 212 (181–246) |  | |
| Mean (SD) | 209 (46) | 216 (50) |  | |
| Duration of chest tube placement, days |  |  | 0.130 | |
| Median (IQR) | 2 (2–2.5) | 2 (2–3) |  | |
| Mean (SD) | 2.6 (1.9) | 3.1 (3.0) |  | |
| Occurrence of prolonged air leak, N (%) | 5 (4.5) | 17 (8.5) | 0.273 | |
| Additional treatment for air leaks, N (%) | 9 (8.1) | 25 (12.6) | 0.302 | |
| Pleurodesis | 5 (4.5) | 19 (9.5) | 0.170 | |
| Reoperation | 1 (0.9) | 5 (2.5) | 0.577 | |
| Chest tube reinsertion or exchange | 6 (5.4) | 7 (3.5) | 0.617 | |
| CI, confidence interval; FEV1, Forced expiratory volume in 1 second; OR, odds ratio; VC, vital capacity | | | |  |

**Supplementary Figure Legend:**

**Figure S1**

**Flowchart of the intraoperative air leak assessment method**.

In the conventional method (which relies solely on the water-sealing test), detected air leaks were managed with soft coagulation, suturing, and reinforcement with fibrin glue and a polyglycolic acid sheet, with the disappearance of the air leak signifying the completion of the operation. The new method incorporates tidal volume measurements and adds to the abovementioned procedures that are performed using the conventional method. However, if the difference between the inspired and expired tidal volumes exceeded 20 mL per ventilation, an air leak was suspected, prompting further examination of the intrathoracic cavity to identify and repair it.
